# Multidetector CT angiography of the Circle of Willis: association of its variants with carotid artery disease and brain ischemia

**DOI:** 10.1007/s00330-018-5577-x

**Published:** 2018-06-19

**Authors:** Andrea Varga, Giovanni Di Leo, Péter Vince Banga, Csaba Csobay-Novák, Márton Kolossváry, Pál Maurovich-Horvat, Kálmán Hüttl

**Affiliations:** 1 Department of Diagnostic Radiology, Heart and Vascular Center, Semmelweis University, 18 Határőr street, Budapest, 1122 Hungary; 2 Radiology Unit, IRCCS Policlinico San Donato, Milan, Italy; 3 Department of Vascular Surgery, Heart and Vascular Center, Semmelweis University, Budapest, Hungary; 4 Department of Interventional Radiology, Heart and Vascular Center, Semmelweis University, Budapest, Hungary; 5 MTA-SE Cardiovascular Imaging Research Group, Heart and Vascular Center, Semmelweis University, Budapest, Hungary

**Keywords:** Circle of Willis, Anatomy, Carotid Artery, Internal, CT Angiography, Endarterectomy, Carotid

## Abstract

**Purpose:**

(1) to estimate the prevalence of Circle of Willis (CoW) variants in patients undergoing carotid endarterectomy, (2) to correlate these variants to controls and (3) cerebral ischemia depicted by computed tomography (CT).

**Materials and methods:**

After Institutional Review Board approval, data of 544 carotid endarterectomy patients (331 males, mean age 69±8 years) and 196 controls (117 males, mean age 66±11 years) who underwent brain CT and carotid CT angiography (CTA) were retrospectively analysed. Two observers independently classified each CoW segment as normal, hypoplastic (diameter <0.8 mm) or non-visualized. Four groups of CoW variants based on the number of hypoplastic/non-visualized segments were correlated with clinical data (ANOVA, χ^2^ and multivariate logistic regression analysis). Intra- and inter-observer agreement was estimated using Cohen κ statistics.

**Results:**

High prevalence of CoW variants (97%) and compromised CoW (81%) was observed in the study group and significant difference was found in the distribution of CoW variants compared to controls (*p*<0.001), internal carotid artery (ICA) stenosis being the only independent predictor of CoW morphology (*p*<0.001). Significant correlation was found between CoW configuration and brain ischemia in the study group (*p*=0.002). ICA stenosis of ≥90% was associated to higher rate of ipsilateral A1 hypoplasia/non-visualization (*p*<0.001). Intra- and inter-observer agreement was from substantial to almost perfect (Cohen κ=0.75–1.0).

**Conclusion:**

Highly variable CoW morphology was demonstrated in patients undergoing endarterectomy compared to controls. Likely compromised CoW in relation to cerebral ischemia was observed in a large cohort of carotid endarterectomy subjects.

**Key points:**

*• CoW variant distribution significantly differed in the study and control groups (p<0.001).*

*• ICA stenosis was the only independent predictor of CoW morphology (p<0.001).*

*• Severely compromised CoW configuration showed significant association with brain ischemia (p=0.002).*

## Introduction

Reduction of blood flow due to severe internal carotid artery (ICA) stenosis requires compensation to maintain sufficient perfusion of the affected vascular territory. The Circle of Willis (CoW) is considered the primary collateral pathway, which may allow blood supply from the contralateral ICA or from the basilar artery [1, 2], depending on the continuity of the anterior and posterior parts of the CoW.

In autopsy studies the prevalence of absent or hypoplastic segments was increased in stroke patients as compared to normal subjects [3, 4]. Some investigators reported that incomplete collateral pathways in symptomatic ICA stenosis were associated with increased risk of infarction [1, 5]. An imaging study showed less CoW variants in strongly selected patients with transient ischemic attack (TIA) or ischemic stroke due to carotid artery disease [6], while other authors found higher prevalence of hypoplastic or non-visualized segments [7–9]. Furthermore, patients who have collaterals supplying the operative side are less prone to perioperative stroke [1] or cerebral ischemia during clamping of the ICA [10–13].

Several articles addressed the CoW anatomy using digital subtraction angiography (DSA) [1], transcranial Doppler ultrasound [5, 14] and 3D time-of-flight (3D TOF) magnetic resonance angiography (MRA) [6, 9–12, 14–23]. However, few reports have been published with multi-detector computed tomography angiography (CTA) [7, 24–29], only one of them focusing on patients with ICA stenosis and comparing them with controls [7].

CTA is a fast, minimally invasive examination with high spatial resolution. It is increasingly used for the preoperative assessment of steno-occlusive carotid disease, providing detailed anatomical depiction of the extracranial and intracranial arteries with a single scan. As data on the CoW variants in carotid artery disease are sparse and conflicting, we aimed to estimate the prevalence of CoW variants in a cohort of 544 carotid endarterectomy patients compared to control subjects and correlate these variants with cerebral ischemia. We hypothesized an association between compromised CoW and ICA stenosis as well as higher likelihood of cerebral infarcts.

## Materials and methods

### Study group

After Institutional Review Board approval, we retrospectively analysed all brain CT and carotid CTA examinations of patients who (1) underwent carotid endarterectomy in our hospital between January 2013 and November 2015 for >70% symptomatic or >80% asymptomatic ICA stenosis, according to the NASCET criteria [30]; and (2) had adequate CT and CTA to evaluate the CoW. Procedures were in accordance with institutional guidelines. To avoid biases, no patient selection was made.

### Control group

Reviewing carotid CTAs performed in our institution from January 2014 to November 2017, we identified all subjects with either negative CTA or minor/mild carotid atherosclerosis to provide a sex-matched control group.

### Brain CT and CTA examinations

All examinations were performed using a 256-slice scanner (Brilliance iCT 256, Philips Healthcare, Best, The Netherlands).

Brain CT was obtained using the following parameters (field of view 200-250 mm, collimation 64×0.625, pitch 0.39, gantry rotation time 400 ms, tube voltage 120 kVp, tube current 120-204 mAs, slice thickness 2 mm, dose-length product 312-626 mGycm).

All CTA examinations were performed from the aortic arch to under the vertex using the following parameters (field of view 180-200 mm, collimation 128×0.625, pitch 0.758, gantry rotation time 330 ms, tube voltage 120 kVp, tube current 76-206 mAs, dose-length product 220-608 mGycm). Bolus tracking technique in the aortic arch was used with 50 ml of iodinated contrast agent (Iomeron 400, Bracco Imaging SpA, Milan, Italy) followed by 40 ml saline bolus, both injected at 5 ml/s through a 18G cannula. Contiguous sections were reconstructed with 0.67-mm slice thickness and 512×512 matrix using hybrid iterative reconstruction technique (iDOSE, Philips Healthcare, Cleveland, OH, USA).

Images were evaluated on a dedicated workstation (IntelliSpace Portal, Philips Healthcare, Best, The Netherlands). Visualization included 3-mm maximum intensity projection slabs parallel and perpendicular to the anterior skull base, providing an overview of the CoW, but final decisions were made on the thin section images.

### Study group brain CT assessment

The presence of any ICA territory infarct at the side of surgery was classified as a positive CT regardless of the infarct’s nature (acute/subacute/chronic or territorial/lacunar/watershed). Conversely, the lack of any detectable infarct in the corresponding ICA territory was regarded as a negative CT result.

### Assessment of the supra-aortic arteries and the CoW

In the study group for each side, ICA stenosis was determined according to the NASCET method [30] and categorized using the following four-point score: 0 when <70%, 1 when 70–89%, 2 when 90–99%, and 3 when occluded. The score of the two sides were summed up (range 0–6).

The CoW was evaluated according to three different approaches both in the study and control groups, as follows:

1. *Each individual segment* was scored as normal (diameter ≥0.8 mm), hypoplastic (<0.8 mm) or non-visualized. Each hypoplasia or non-visualization was than classified as single variant or combined with other variants of the CoW. We considered the AcomA as patent if the junctions of A1 and A2 segments were in close contact and, therefore, not separable from each other on the CTA. The communication of the PcomA with both the ICA and the posterior cerebral artery had to be visualized for defining the PcomA;
2. *The anterior and two posterior parts of the CoW* were considered separately. For the anterior part, both A1 segments and AComA were evaluated, since all vessels should be sufficiently developed to allow collateral supply from the contralateral to the ipsilateral ICA. For the posterior part, the P1 segment and the PComA were assessed on each side. Data on the anterior and posterior parts were crossed in a contingency table showing all observed combinations of the CoW (mirror configurations were not considered as separate entities). Moreover, the anterior and the two posterior parts were classified as complete (all segments normal), hypoplastic (any of the components hypoplastic) or incomplete (any of the segments non-visualized);
3. *Four reclassified groups* based on the number of hypoplastic and non-visualized segments were defined, as follows I) complete CoW or only one hypoplastic segment (not/minimally compromised CoW); II) ≥2 hypoplastic segments; III) one non-visualized segment; IV) ≥2 non-visualized segments (severely compromised CoW). Groups II), III) and IV) together were defined as “compromised CoW”.

### Reproducibility of CTA

To estimate the inter-observer agreement in defining CoW morphology on CTA, each individual study subject was assessed by two independent radiologists (R1 with 13 years of experience in vascular imaging; R2, with 8 years of experience), unaware of patient characteristics. In case of discrepancy, agreement was reached by consensus. Intra-observer agreement was evaluated for both observers by comparing data of 100 randomly selected patients at two different reading sessions at least 2 months apart.

### Statistical analysis

Continuous variables were expressed as mean±standard deviation (SD), while categorical variables as counts and percentages. Distributions were given according to the three different approaches to classify the CoW; moreover, variants of the anterior part of the CoW were crossed with those of the posterior part. Bivariate association analysis was performed using the ANOVA for continuous variables or using the χ^2^ test for categorical variables. The multiplicity of statistical tests accounts for applying the Bonferroni method. Considering that a statistical test was performed for each of the 11 analysed variables, the threshold for significance was divided by 10 and defined as *p*≤0.005. All variables that were significantly different between study patients and controls at bivariate analysis were entered into a multivariate logistic regression analysis for ordinal data.

In the χ^2^ test used for brain CT analysis *p*≤0.05 was considered statistically significant.

Intra- and inter-observer agreement was estimated using the Cohen κ statistics. Cohen’s κ values were interpreted as: 0.81–1.00, excellent; 0.61–0.80, good.

All statistical calculations were performed using SPSS software (SPSS v.23; IBM Corp., Armonk, NY).

## Results

### Characteristics of the study and control groups

From 898 consecutive carotid endarterectomy patients in the study period, we excluded 354 patients (39%) due to poor image quality (24/898, 3%) or missing/incomplete CTA (330/898, 36%). The remaining 544 study subjects were analysed (331 males, mean age 69±8 years, range 44–90 years). Of them, 205 (38%) had symptomatic ICA stenosis, including 59 patients (11%) with previous minor stroke, 25 (5%) with amaurosis fugax and 121 (22%) with TIA. The three study subjects with stenosis of <70% were all symptomatic.

A total of 196 control subjects were analysed (117 males, mean age 66±11 years, range 37–93 years). The indication for CTAs was: (1) false positive ultrasound scan in 58 cases (30%); (2) brachiocephalic/subclavian artery stenosis or aneurysm in 27 (14%); (3) cardiology referral in 29 (14.5%); (4) diagnostic work-up before vascular intervention/surgery in 15 (7.5%); (5) neurology referral in 56 (29%); (6) carotid artery dissection in five (2%); (7) neck tumor in four (2%); and (8) vascular malformation in two (1%).

Further details are reported in Table 1.

**Table 1:** Demographics, cardiovascular risk factors, degree of internal carotid artery stenosis and different configurations of Circle of Willis in 544 study subjects and 196 control subjects

	Study group (*N*=544)	Control group *(N*=196)	*p*-value
***Demographics***			
Male gender	331 (61%)	117 (60%)	0.777
Mean age ± SD (years)	69 ± 8	66±11	<0.001
Symptomatic	205 (38%)	-	-
***Cardiovascular risk factors, N (%)***		***Data available in 173 patients***	
Hypertension	500 (92%)	110 (64%)	<0.001
Cigarette smoking	175 (32%)	16 (9%)	<0.001
Hyperlipidemia	234 (43%)	49 (28%)	0.001
Coronary artery disease	170 (31%)	30 (17%)	<0.001
Chronic pulmonary disease	53 (10%)	13 (8%)	0.377
Chronic kidney disease (Stage IIIb-V)	16 (3%)	6 (3%)	0.726
Diabetes	203 (37%)	32 (18%)	<0.001
***Carotid artery stenosis, N (%)***			
<70% on both sides (Score 0)	3 (0.6%)	-	-
70–89% on the side of surgery (Score 1)	128 (23.5%)	-	-
70–89% on both sides (Score 2)	26 (4.8%)	-	-
90–99% on the side of surgery (Score 2)	282 (51.7%)	-	-
90–99% on the side of surgery + 70–89% on the contralateral side (Score 3)	54 (9.9%)	-	-
90–99% on both sides (Score 4) or 70–89% on the side of surgery + contralateral occlusion (Score 4) or 90–99% on the side of surgery + contralateral occlusion (Score 5)	51 (9.4%)	-	-
***CoW groups N (%)***			
Group I)	78 (14%)	55 (28%)	<0.001
Group II)	97 (18%)	52 (27%)	
Group III)	191 (35%)	55 (28%)	
Group IV)	178 (33%)	34 (17%)	

*CoW =* Circle of Willis*; SD =* standard deviation

#### Study group analysis

##### Brain CT analysis

At CT 146/544 study subjects (27%) had a detectable recent or old infarct in the territory of the operated ICA. As shown by the χ^2^ test, the prevalence of brain ischemia was significantly higher (*p*=0.002) in subjects with severely compromised CoW (35%) as compared to the pooled groups I)-III) (23%). Table 2. As a recent brain infarct larger than 1/3 of the corresponding ICA territory is a contraindication for carotid endarterectomy, we have not found major recent infarction in any of our study patients.

**Table 2:** Demographics, cardiovascular risk factors, degree of internal carotid artery stenosis and percentage of cerebral ischemia in different configurations of Circle of Willis in 544 study subjects

	Group I) Complete CoW or 1 hypoplastic segment	Group II) ≥2 hypoplastic segments	Group III) 1 non-visualized segment	Group IV) ≥2 non-visualized segments	*p*-value
***Demographics***	(*n* = 78)	(*n* = 97)	(*n* = 191)	*(n* = 178)	
Male gender	48 (61.5%)	51 (52.6%)	121 (63.4%)	111 (62.4%)	0.324
Age ± SD (years)	68 ± 9	67 ± 8	69 ± 8	70 ± 8	0.107
Symptomatic	21 (26.9%)	38 (39.2%)	68 (35.6%)	78 (43.8%)	0.067
***Cardiovascular risk factors, N (%)***					
Hypertension	70 (89.7%)	89 (91.8%)	175 (91.6%)	166 (93.2%)	0.813
Cigarette smoking	26 (33.3%)	39 (40.2%)	73 (38.2%)	37 (20.8%)	0.001
Hyperlipidemia	41 (47.5%)	41 (42.3%)	77 (40.3%)	75 (43.9%)	0.315
Coronary artery disease	34 (43.6%)	31 (32.0%)	52 (27.2%)	53 (29.8%)	0.067
Chronic pulmonary disease	9 (8.9%)	14 (14.4%)	19 (9.9%)	11 (7.1%)	0.290
Chronic kidney disease (Stage IIIb to V)	2 (2.0%)	5 (5.2%)	4 (2.1%)	5 (3.2%)	0.472
Diabetes	26 (31.7%)	36 (37.1%)	69 (36.1%)	72 (42.3%)	0.707
***ICA stenosis, N (%)***					
<70% on both sides (Score 0)	0 (1.0%)	0 (0.0%)	0 (0.0%)	3 (1.3%)	0.010
70–89% on the side of surgery (Score 1)	13 (20.8%)	20 (20.6%)	52 (27.2%)	43 (22.6%)	
70–89% on both sides (Score 2)	5 (5.9%)	3 (3.1%)	8 (4.2%)	10 (5.8%)	
90–99% on the side of surgery (Score 2)	35 (43.6%)	59 (60.8%)	94 (49.2%)	94 (54.8%)	
90–99% on the side of surgery + 70–89% on the contralateral side (Score 3)	12 (13.9%)	5 (5.2%)	14 (7.3%)	23 (13.5%)	
90–99% on both sides (Score 4) or 70–89% on the side of surgery + contralateral occlusion (Score 4) or 90–99% on the side of surgery + contralateral occlusion (Score 5)	13 (14.9%)	10 (10.3%)	23 (12.0%)	5 (1.9%)	
***Brain CT N (%)***					
Negative 398 (73%)	61 (15%)	74 (19%)	151 (38%)	112 (28%)	0.002*
Positive 146 (27%)	19 (13%)	24 (16%)	42 (29%)	61 (42%)	

*SD =* standard deviation; *ICA =* internal carotid artery

*χ^2^ test between pooled Groups I-III versus Group IV

##### Analysis of all individual segments

The number and frequency of normal, hypoplastic or non-visualized segments (single variant or mostly combined with others) are presented in Table 3. Non-visualized PComA (447/1088, 41.1%), AComA hypoplasia (154/544, 28.3%) and PComA hypoplasia (275/1088, 25.3%) were the most frequent.

**Table 3:** Number and frequency of normal, hypoplastic or non-visualized/incomplete individual segments and anterior and posterior parts of the Circle of Willis of 544 study subjects and 196 control subjects

Segment/Circle		STUDY GROUP (*N*=544)			CONTROL GROUP (*N*=196)			p*
		Normal	Hypoplasia	Non-visualization	Normal	Hypoplasia	Non-visualization	
**AComA (N)**	All	369 (67.8%)	154 (28.3%)	21 (3.9%)	155 (79.1 %)	40 (20.4%)	1 (0.5%)	0.003
	Single variant	N/A	9 (1.7%)	2 (0.4%)				
	Combined variant	N/A	145 (26.7%)	19 (3.5%)				
**A1** **(N x 2)**	All	964 (88.6%)	81 (7.4%)	43 (4.0%)	380 (97.0%)	8 (2.0%)	4 (1.0%)	<0.001
	Single variant	N/A	6 (0.6%)	2 (0.2%)				
	Combined variant	N/A	75 (6.9%)	41 (3.8%)				
**PComA** **(N x 2)**	All	366 (33.6%)	275 (25.3%)	447 (41.1%)	161 (41.1%)	121 (30.9%)	110 (28.0%)	0.008
	Single variant	N/A	36 (3.3%)	26 (2.4%)				
	Combined variant	N/A	239 (22.0%)	421 (38.7%)				
**P1** **(N x 2)**	All	948 (87.1%)	81 (7.4%)	59 (5.4%)	354 (90.3%)	33 (8.4%)	5 (1.3%)	0.098
	Single variant	N/A	6 (0.6%)	2 (0.2%)				
	Combined variant	N/A	75 (6.9%)	57 (5.2%)				
**Anterior CoW** **(N)**	All	257 (47.2%)	223 (41.0)	64 (11.8%)	143 (73.0%)	48 (24.5%)	5 (2.5%)	<0.001
	Complete posterior part	19 (3.5%)	198 (4.5%)	60 (11.0%)				
	Posterior part variant	238 (43.8%)	25 (36.4%)	4 (0.7%)				
**Posterior CoW** **(N x 2)**	All	234 (21.9%)	351 (32.2%)	503 (46.2%)	123 (31.4%)	154 (39.3%)	115 (29.3%)	<0.001
	Complete anterior part	38 (3.5%)	182 (16.5%)	226 (20.8%)				
	Anterior part variant	196 (18.0%)	169 (15.5%)	277 (25.5%)				

*CoW =* Circle of Willis; *AComA =* anterior communicating artery; *A1 =* precommunicating segment of the anterior cerebral artery; *PComA =* posterior communicating artery; *P1 =* precommunicating segment of the posterior cerebral artery; *N/A =* not applicable

*Comparison between study subjects and controls, pooling hypoplasia and non-visualization versus normal

The frequencies of non-visualization and hypoplasia of each CoW segment reported in anatomic and imaging studies as well as those found by us are summarized in Table 4.

**Table 4:** Frequency of non-visualized and hypoplastic individual segments and incomplete and hypoplastic anterior and posterior Circles of Willis in autopsy and imaging studies

Segment/circle		Autopsy studies [4, 31–34]	Imaging studies		Present Study	
			Controls	Study group	Controls	Study group
**AComA**	Non-visualization	0–3.2%	1–19% [7, 22, 23, 28, 29]	4–40% [6, 7, 10, 18, 20, 21, 26]	3.9%	0.5%
	hypoplasia	3–32%	23% [29]	4–11% [20, 24]	28.3%	20.4%
**A1**	Non-visualization	0–0.8%	1–7% [7, 22, 23, 28, 29]	4–15% [6, 7, 10, 18, 20, 21, 24–26]	4.0%	1.0%
	hypoplasia	1.5–7.5%	4–10% [7, 28, 29]	8–24% [7, 10, 24, 26]	7.4%	2.0%
**PComA**	Non-visualization	0–3.5%	22–38% [7, 9, 22, 23, 28, 29]	21–66% [6, 7, 10, 12, 18–21, 24–27]	41.1%	28.0%
	hypoplasia	23–70%	38–41% [7, 29]	6–18% [7, 20, 24]	25.3%	30.9%
**P1**	Non-visualization	0–2.4%	0–2% [7, 22, 23, 28, 29]	3–10% [6, 7, 10, 20, 24, 26]	5.4%	1.3%
	hypoplasia	12–23%	3–6% [7, 28, 29]	1–8% [6, 7, 18, 20, 24]	7.4%	8.4%
**Anterior CoW**	incomplete	-	2–12% [7, 8, 23, 28, 29]	12–24% [7, 10, 12, 25]	11.8%	2.5%
	hypoplastic	-	4–10% [7, 8, 28]	17% [8, 12]	41.0%	24.5%
**Posterior CoW**	incomplete	-	23–39% [23, 28, 29]	37–74% [6, 8, 10, 12, 21, 25]	46.2%	29.3%
	hypoplastic	-	29% [28]	15–40% [8, 12]	32.2%	39.3%

The numbers in brackets are the references.

*AComA* = anterior communicating artery, *A1* = precommunicating segment of the anterior cerebral artery, *CoW* = Circle of Willis, *PComA* = posterior communicating artery, *P1* = precommunicating segment of the posterior cerebral artery

##### Analysis of the anterior, posterior parts and the entirety of the CoW

The frequency and number of all possible combinations of the anterior and posterior variants are listed in Table 5. The variants of the anterior part fell into five types. The posterior part of the CoW demonstrated higher variability and was classified into 16 groups. Bilateral non-visualization of the PComA (118/544, 21.7%), combined non-visualization of one PComA and hypoplasia of the other PcomA (93/544, 17.1%) were the most frequent.

**Table 5:** Circle of Willis configurations as a combination of the anterior and posterior variants in 544 study subjects

Both posterior CoWs		Anterior CoW					
		complete	AComA hypoplasia	AComA non-visualization	A1 hypoplasia	A1 non-visualization	Total
As a whole	complete	19 (3.5%)	9 (1.7%)	2 (0.4%)	7 (1.3%)	2 (0.4%)	39 (7.3%)
PComA	unilateral hypoplasia	35 (6.4%)	12 (2.2%)	0	12 (2.2%)	5 (0.9%)	64 (11.7%)
	unilateral non-visualization	26 (4.8%)	20 (3.7%)	2 (0.4%)	10 (1.8%)	9 (1.7%)	67 (12.3%)
	bilateral hypoplasia	25 (4.6%)	9 (1.7%)	1 (0.2%)	8 (1.5%)	3 (0.6%)	46 (8.4%)
	bilateral non-visualization	44 (8.1%)	39 (7.2%)	10 (1.8%)	19 (3.4%)	6 (1.1%)	118 (21.7%)
	non-visualization - contralateral hypoplasia	46 (8.4%)	27 (5.0%)	1 (0.2%)	11 (2.0%)	8 (1.5%)	93 (17.1%)
PComA-P1	PComA hypoplasia - contralateral P1 hypoplasia	6 (1.1%)	4 (0.7%)	0	2 (0.4%)	1 (0.2%)	13 (2.4%)
	PComA and contralateral P1 non-visualization	13 (2.4%)	6 (1.1%)	0	1 (0.2%)	2 (0.4%)	22 (4.0%)
	PcomA hypoplasia - contralateral P1 non-visualization	7 (1.3%)	1 (0.2%)	0	0	2 (0.4%)	10 (1.8%)
	PcomA non-visualization - contralateral P1 hypoplasia	13 (2.4%)	5 (0.9%)	2 (0.4%)	2 (0.4%)	0	22 (4.0%)
P1	unilateral hypoplasia	7 (1.3%)	5 (0.9%)	0	7 (1.3%)	1 (0.2%)	20 (3.7%)
	unilateral non-visualization	2 (0.4%)	0	1 (0.2%)	1 (0.2%)	1 (0.2%)	5 (0.9%)
	bilateral hypoplasia	2 (0.4%)	2 (0.4%)	0	0	1 (0.2%)	5 (0.9%)
	bilateral non-visualization	3 (0.6%)	0	1 (0.2%)	0	1 (0.2%)	5 (0.9%)
	non-visualization - contralateral hypoplasia	6 (1.1%)	2 (0.4%)	0	0	1 (0.2%)	9 (1.7%)
Other	multiple hypoplasia and/or non-visualization	3 (0.6%)	2 (0.4%)	1 (0.2%)	0	0	6 (1.1%)
Total		257 (47.0%)	143 (26.5%)*	21 (3.9%)	80 (14.7%)	43 (8.0%)	544 (100%)

*AComA =* anterior communicating artery; *A1 =* precommunicating segment of the anterior cerebral artery; *CoW =* circle of Willis; *PComA* = posterior communicating artery; *P1 =* precommunicating segment of the posterior cerebral artery

*11 AcomA hypoplasia was combined with 10 cases of A1 hypoplasia and one A1 non-visualization counted as A1 variants to avoid duplications

Only 19/544 study subjects had an entirely complete CoW (3.5%).

**Fig. 1:**
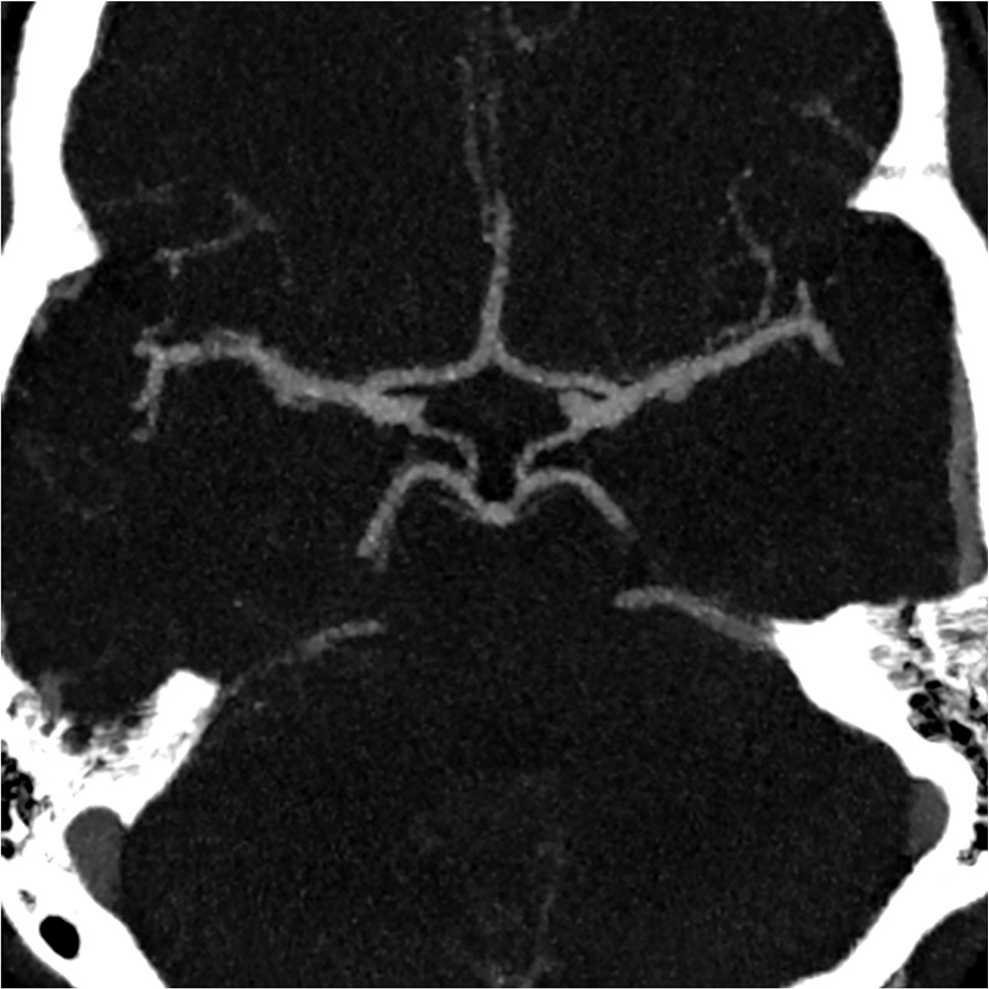
Thick slab maximum intensity projection (7 mm) of the Circle of Willis showing a normal circle with all components ≥0.8 mm (group I)

**Fig. 2:**
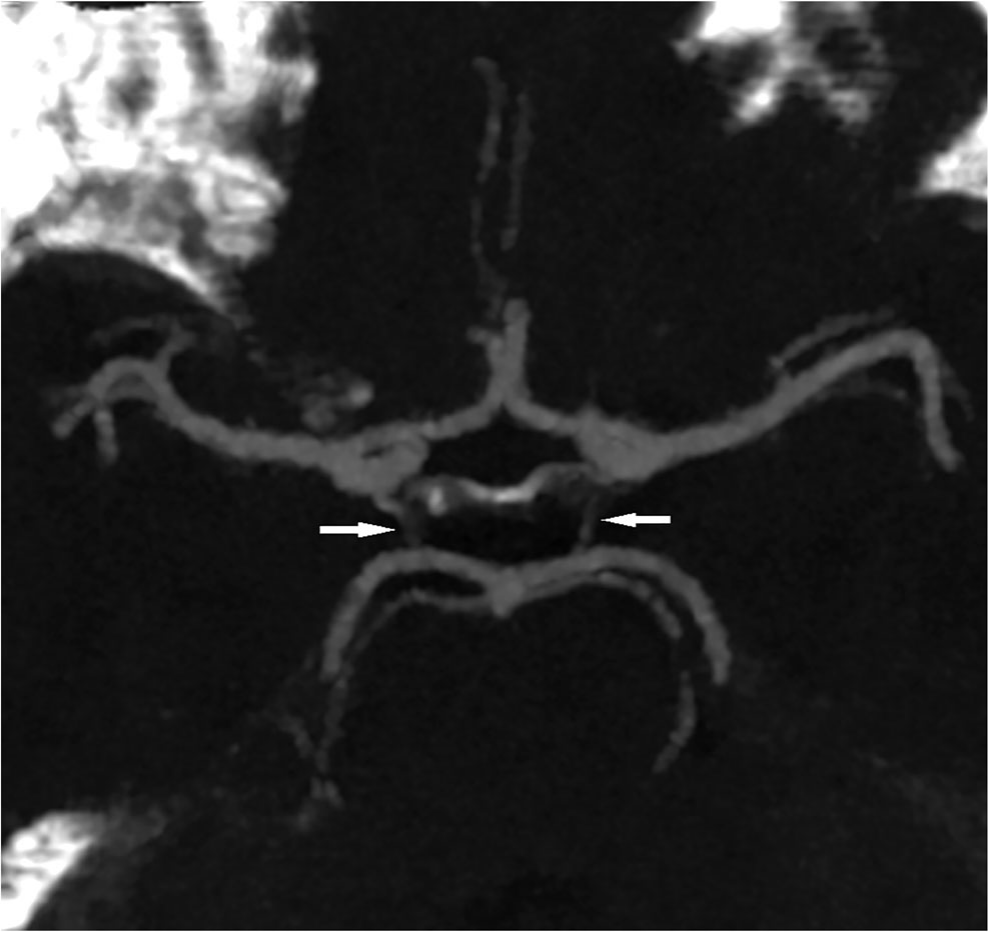
Thick slab maximum intensity projection (10 mm) showing bilateral hypoplasia (<0.8 mm) of the posterior communicating artery (arrows) (group II)

**Fig. 3:**
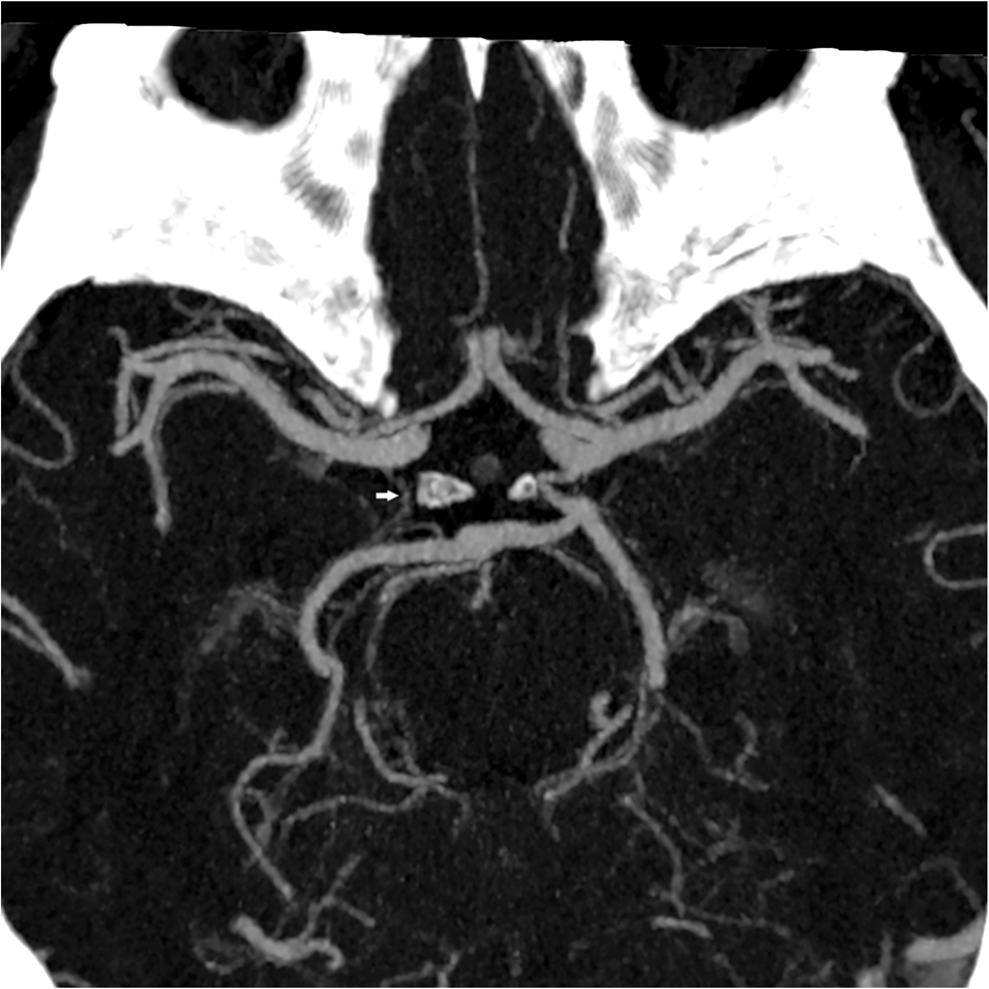
Thick slab maximum intensity projection (10 mm): the right posterior communicating artery is not visualized. The filiform vascular structure filled with contrast along its expected course is the vein of Rosenthal (arrow) (group III)

**Fig. 4:**
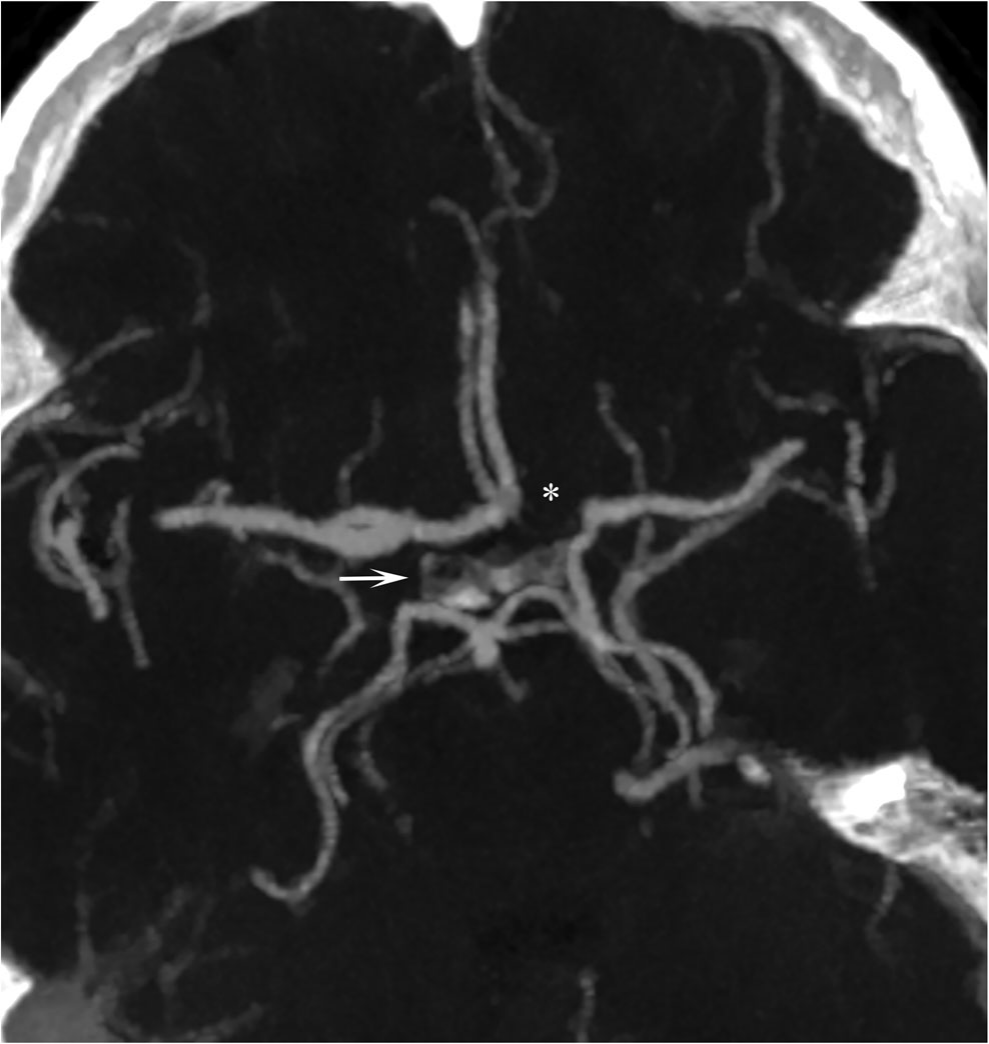
Thick slab maximum intensity projection (8 mm): neither the left precommunicating segment of the anterior cerebral artery (asterics) nor the right posterior communicating artery (arrow) is visualized (group IV)

Examples of CoW groups I)-IV) are shown in Figs. 1, 2, 3, and 4.

##### Correlation analysis

The association of CoW configurations and demographics, cardiovascular risk factors, and degree of ICA stenosis is detailed in Table 2. Groups II), III) and IV) together represented 81% (443/544) of our study group.

After Bonferroni correction, the association of CoW configuration and ICA stenosis did not reach the pre-specified significance level (*p*=0.010). Unilateral stenosis of 90-99% (Score 2) was the most frequent in all the four CoW configurations, ranging from 44% to 61%. Notably, high carotid stenosis scores (Scores 4 and 5) were the least frequent in group IV). Considering each single CoW segment, ICA stenosis of the side of surgery was correlated only to ipsilateral A1 segment (*p<*0.001). Hypoplastic/non-visualized ipsilateral A1 segment was present in 81/370 (22%) of patients with an ICA stenosis of ≥90% while only in 14/174 (8%) of patients with a stenosis of <90%.

The percentage of smokers was the lowest in patients with severely compromised CoW. The other comparisons showed no significant difference.

##### Reproducibility of the CTA

The inter-observer agreement in the assessment of the AComA was good (*κ*=0.75) while all other κ values were excellent (intra-observer agreement *κ*=0.84-1.0; inter-observer agreement *κ*=0.82–0.92).

We evaluated 3808 (544×7) segments and encountered inter-observer discrepancy in 212 cases (5.6%), mostly for the PcomA (98/212, 46.2%), followed by the AComA (60/212 28.3%). These were mainly one-category discrepancies (hypoplasia versus normal/non-visualization) in 196/212 (92.5%).

#### Control group analysis

##### Analysis of all individual segments

Hypoplasia of PComA (121/392, 30.9%), non-visualization of PComA (110/392, 28.0%) and hypoplasia of AComA (40/196, 20.4%) were the most frequent variants in control subjects (Table 3). Non-visualization of the A1 and AComA was rare, 4/392 (1.0%) and 1/196 (0.5%), respectively.

##### Analysis of the anterior, posterior parts and the entirety of the CoW

Amongst controls, the most common posterior variants were unilateral PComA hypoplasia (43/196, 21.9%), bilateral PComA hypoplasia (41/196, 20.9%), bilateral non-visualization of the PComA (31/196, 15.8%), combined non-visualization of one PComA and hypoplasia of the other PcomA (29/196, 14.8%) and unilateral non-visualization of PComA (24/196, 12.2%).

Of 196 control subjects, 21 (11%) had an entirely complete CoW.

#### Comparison of the study and control groups

At bivariate analysis (Table 1), the study subjects and controls were different in terms of five cardiovascular risk factors as well as in terms of coronary artery disease (*p*<0.001 for all). Moreover, they differed in CoW variant distribution (*p*<0.001). However, the multivariate logistic regression analysis showed that the ICA stenosis was the only independent predictor of CoW morphology (*p*<0.001). Not or minimally compromised CoW and severely compromised CoW groups accounted for 14% and 33% of the study group, whereas an opposite trend was found in controls with 28% and 17%, respectively.

The same difference was encountered with the analysis of each individual segments and the anterior/posterior parts of the CoW confirming a significantly lower rate of hypoplasia or non-visualization (*p*≤0.008) in controls except for the P1 (Table 3).

## Discussion

Our major findings were the significantly higher prevalence of CoW variants and the association of CoW configuration with brain ischemia in the study group, underlining the importance of recognition of these variants for surgical and endovascular treatment planning.

Based on unselected post-mortem studies, the prevalence of normal CoW ranges from 15% to 59% [3, 4, 31–34]. In imaging studies completeness of the entire CoW was reported in 27%–90% of healthy individuals [7, 22, 28, 29] and 18%–55% in cerebrovascular diseases [6, 21, 25]. The different criteria used to define a normal or incomplete CoW can account for the variability in these data. The prevalence of a complete CoW was 11% in controls and even lower in our study group (3.5%) using a firm definition of completeness (all components ≥0.8 mm) as a prerequisite for development of collateral flow.

### Comparison of patients’ CoW variants with autopsy and imaging studies

The non-visualization of any segment and the hypoplasia of A1 were more frequently reported in cerebrovascular patients as compared to healthy subjects, with some overlap between the two groups, while an opposite trend was demonstrated with respect to AComA and PComA hypoplasia (Table 4).

Comparing our study subjects only with CTA studies on cerebrovascular patients, the prevalence of non-visualized AComA (4%), A1 (4%) and the most commonly compromised PcomA (41%) in our analysis was less frequent (versus 6%-15% and 47%–66%, respectively).

The fact that CTA does not depend on flow velocity (as opposed to 3D TOF MRA) and the higher spatial resolution achieved by 256-slice CT might have contributed to the lower prevalence of non-visualized AComA, A1 and PComA. However, a certain percentage of the non-visualized segments in this study could have been hypoplastic vessels below the CTA detection capability, as in autopsy studies absence was found rarely (0%–3.5%).

Comparing our study group analysis with imaging studies on cerebrovascular patients, we found a higher prevalence of AComA and PcomA hypoplasia (28% and 25%, respectively). The higher detection rate of hypoplastic communicant arteries thanks to the higher spatial resolution of the 256-slice CT relative to the 16-40-row scanners or the MRA techniques used by other investigators might partly account for this difference.

Discontinuity of the CoW in patients with symptomatic ICA stenosis was associated with higher risk of TIA and ischemic stroke [1]. Subjects with high-grade ICA stenosis or occlusion with nil or only one ipsilateral collateral vessel (A1, PComA) had a higher likelihood of stroke when compared to patients with two functional ipsilateral collaterals [5]. This is in agreement with the higher prevalence of brain ischemia found in our patients with severely compromised CoW (*p*=0.002). We must acknowledge, however, that CT has low sensitivity in the detection of recent ischemia relative to diffusion-weighted magnetic resonance (MR)  imaging but only brain CT was performed for the vast majority of the study group according to institutional protocols with only 11 study subjects having concurrent brain MR imaging and CT.

Most of the previous imaging studies on CoW variants found higher prevalence of hypoplastic or non-visualized segments in cerebrovascular patients [5, 7–9] in accordance with our study. The anterior part of the CoW is generally considered the most important route for collateral flow in severe ICA stenosis [5–7, 18, 35]. Kluytman et al. found that the PComA alone had little compensating capacity in ICA occlusion. Best-preserved hemodynamics was reported if both the anterior and posterior parts of the CoW were recruited [35].

The high frequency of compromised circles and the clear difference between the study and control groups strongly suggests that our study subjects have fewer functional segments hindering hemodynamic adaptation. Although the study group and controls were significantly different in terms of five cardiovascular risk factors and coronary artery disease, the multivariate logistic regression analysis showed that ICA stenosis was the only independent predictor of CoW morphology (*p*<0.001). The higher frequency of ipsilateral A1 hypoplasia/non-visualization was positively associated to ipsilateral ICA stenosis of ≥90% (*p<*0.001), also implying a correlation between carotid artery disease and hindered collateral recruitment.

Our research group also found significant association between combined incomplete anterior and ipsilateral posterior parts of the CoW and immediate postoperative neurologic complications after carotid endarterectomy without shunt protection (31% vs 5%, *p*<0.001) [13]. This is in line with another study, in which ≥2 non-visualized segments versus complete CoW were shown to have a statistically significant risk to develop carotid clamping intolerance [11]. In another study, in case of contralateral ICA occlusion, the risk of intraoperative TIA was significantly increased when both parts of the CoW (in particular the posterior part) were incomplete [12].

Three limitations of our study were the lack of comparison with DSA as reference of standard, the possible bias between the study and control groups and the fact that the study group was limited to patients eligible for carotid endarterectomy with either asymptomatic ICA stenosis or symptomatic stenosis with TIA, amaurosis fugax or minor ischemic stroke. DSA and diffusion-weighted MR imaging are not routinely performed as CTA has taken over the role of DSA in the preoperative assessment of the carotid arteries in our institution, moreover it can be easily performed together with brain CT. Further investigations with a well-matched control group including diffusion-weighted MR imaging are needed.

In conclusion, highly variable CoW morphology compared to controls was demonstrated and a likely compromised CoW in relation to ICA stenosis and brain ischemia was observed in a large cohort of carotid endarterectomy subjects.

## Abbreviations

3D TOF MRA: 3D time-of-flight MRA
A1: Precommunicating segment of the anterior cerebral artery
AComA: Anterior communicating artery
CoW: Circle of Willis
CTA: Computed tomography angiography
DSA: Digital subtraction angiography
ICA: Internal carotid artery
MRA: Magnetic resonance angiography
P1: Precommunicating segment of the posterior cerebral artery
PComA: Posterior communicating artery
TIA: Transient ischemic attack
